# Management of Oehler's Type III Dens Invaginatus Using Cone Beam Computed Tomography

**DOI:** 10.1155/2016/3573612

**Published:** 2016-03-16

**Authors:** Jaya Ranganathan, Mohan Kumar Rangarajan Sundaresan, Srinivasan Ramasamy

**Affiliations:** Department of Conservative Dentistry and Endodontics, Priyadarshini Dental College & Hospital, Pandur, Thiruvallur, Chennai 631203, India

## Abstract

Dens Invaginatus is a dental malformation that poses diagnostic difficulties in the clinical context. This anomaly may increase the risk of pulp disease and can potentially complicate endodontic procedure due to the aberrant root canal anatomy. Compared to conventional radiographs, three-dimensional images obtained with Cone Beam Computed Tomography (CBCT) are invaluable in the diagnosis of the extent of this anomaly and in the appropriate treatment planning. Oehler's classification (1957) for Dens Invaginatus (DI) into three types depending on the depth of the invagination has been used for treatment planning. Of the three types Type III DI is characterized by infolding of the enamel into the tooth up to the root apex and is considered as the most severe variant of DI and hence the most challenging to treat endodontically, due to the morphological complexities. This report describes a case of Oehler's Type III DI in a necrotic permanent maxillary lateral incisor in which CBCT images played a key role in diagnosis and treatment planning. The case was managed successfully by a combination of nonsurgical and surgical endodontic therapy with orthograde and retrograde thermoplastic gutta percha obturation.

## 1. Introduction

Dens Invaginatus is a developmental anomaly which arises as a result of an invagination into the surface of the tooth crown before calcification has occurred [1].

Dens Invaginatus (DI) in a human tooth was first described by Socrates in 1856 [2]. The term Dens Invaginatus was proposed by Oehler in 1957 and is the most widely accepted. Synonyms for this anomaly reported in literature are dens in dente (Busch 1897), invaginated odontome, dilated gestant odontome, dilated composite odontome (Hunter 1951), tooth inclusion, telescopic tooth, and gestant anomaly [2–5].

The teeth most affected are the maxillary lateral incisors. Bilateral occurrence as high as 43% has been reported by Hülsmann [2]. There are rare case reports of DI in the posterior, deciduous, and supernumerary teeth [2, 3, 6]. The reported prevalence of adult teeth affected by DI is between 0.3 and 10%. This variation in reported prevalence is attributed to methodological differences in cohort studies, the identification criteria used, and diagnostic difficulties [2, 6, 7].

The most widely accepted classification of this anomaly is that proposed by Oehler (1957) who categorized it based on the enamel invagination depth observed radiographically. Type I describes an enamel lined invagination confined to the coronal part of the tooth; Type II represents extension of the invagination beyond the cementoenamel junction ending as a blind sac. Type III includes permeation of the root by the invagination to form an additional canal opening on lateral side of root [8]. This invaginated tract which usually has no direct communication with the pulp has been termed pseudo canal by Gonçalves et al. [4]. The invagination may be completely lined with enamel but frequently cementum may be found lining the invagination in the apical portion [2]. Amongst the three types Type I and Type II are considered as incomplete invagination and their incidence is 79% and 15%, respectively. Type III as reported in the present case is a complete invagination with the lowest incidence at 5% [3, 6].

There is a lack of consensus on the aetiology of DI. Various proposed theories are embryological cause with proliferation of enamel organ cells into dental papilla (Rushton 1937), retardation of focal group of cells (Kronfeld 1934), external forces (Atkinson 1943), trauma (Gustafson & Sundberg 1950), infection (Fischer 1936), and genetic factors as reported by Hülsmann, Alani, and Coriani [2, 3, 6].

Even though DI is one of the most common developmental anomalies, it is easily overlooked as very often there is absence of significant clinical signs of the anomaly and it is only detected on routine radiographs. Clinically, an unusual crown morphology (dilated/peg shaped), grooving of the palatal enamel, exaggerated/bifid cingulum, or a deep foramen cecum may be hints of the anomaly though the affected tooth may show no obvious clinical signs of the malformation. Due to the high incidence of bilateral occurrence, the contralateral tooth should also be investigated [2, 9]. The presence of DI predisposes the entry of bacteria into the invagination. If undetected in the early stages this leads to pulp/periodontal pathosis as there are channels between the invagination and the pulp space. The presence of hypomineralized enamel surrounding the invagination predisposes to early involvement of the pulp space through the invagination [2, 4, 5, 7, 10–12].

Clinically, severe pulpal (loss of vitality) and periodontal alterations as well as presence of sinus tracts can be associated with an untreated DI with pulp involvement [13].

Conventional radiographs are insufficient in most cases of DI as they show only a 2D view of a complex anatomy. The use of CBCT (Cone Beam Computed Tomography) images as was done in our case report, obtained in 3 orthogonal planes (axial, sagittal, and coronal), is invaluable to observe the extent of the invagination and the relationship of the invaginated portion and the root canal with high precision [6, 13, 14]. The use of CBCT images was crucial in our treatment planning.

Treatment of a tooth with DI and pulp pathosis is a challenge to an endodontist owing to the irregular shape of the root canal systems and invaginated tract [2]. The problems are further compounded if part of the invagination is obliterated or if the invagination encroaches into the pulp space at some level as was also observed in our case.

This case report describes Oehler's Type III DI in a necrotic permanent maxillary lateral incisor with periapical lesion managed successfully with a nonsurgical and surgical endodontic therapy with orthograde and retrograde backfilling of thermoplastic gutta percha. The use of CBCT images in this case was vital in diagnosis and treatment planning.

## 2. Case Presentation

A 14-year-old female reported to the dental hospital with complaints of pain and occasional swelling in relation to her right upper front tooth for past 6 months. Pain was aggravated by taking hot foods and mastication. History revealed that maxillary right central incisor was “filled” eight months earlier in a private dental hospital but no relevant treatment details or documents were available. Medical history was noncontributory.

**Figure 1:**
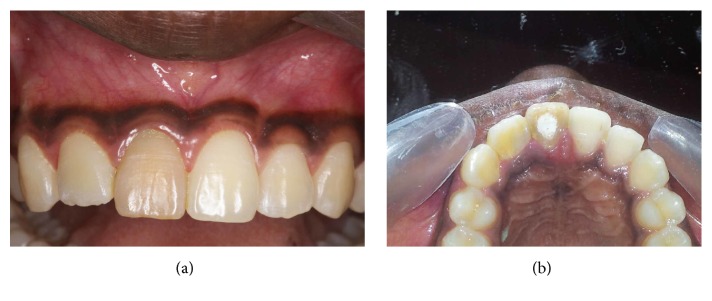
(a) Labial view. (b) Palatal view.

Intraoral examination revealed a slight discoloration of the maxillary first central incisor and a diffuse swelling of the vestibular region adjacent to maxillary right central and lateral incisor. There was pain on percussion. The mesiodistal width of the maxillary right lateral incisor was slightly larger than that of the contralateral tooth; this difference was more prominent in the palatal view (Figures 1(a) and 1(b)).

Pulp sensibility tests of the tooth (thermal test with heated gutta percha and electric pulp testing (Parkell Electronics Division, Farmingdale, NY)) revealed irreversible pulpitis of maxillary central and lateral incisors.

**Figure 2:**
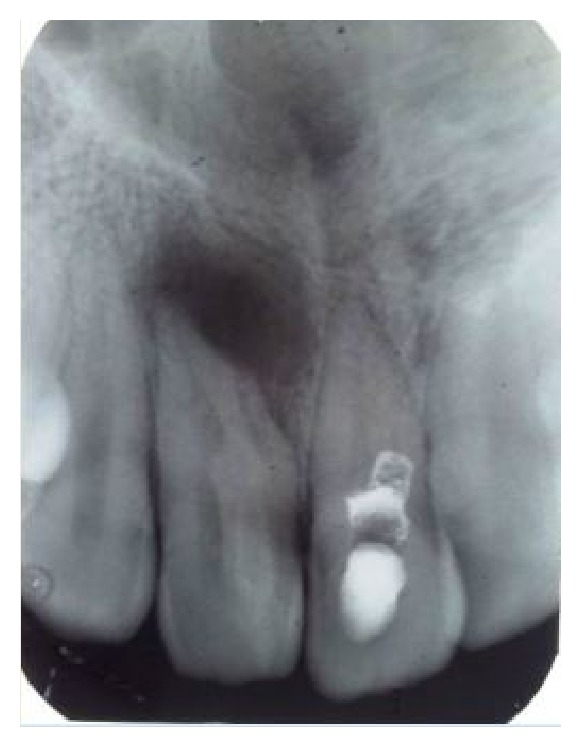
Intraoral periapical image.

**Figure 3:**
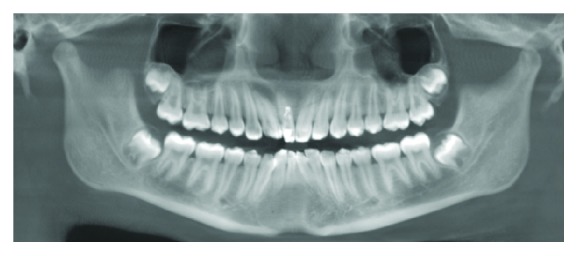
OPG.

On radiological examination (intraoral periapical and OPG) a radioopaque material was seen in coronal part of maxillary right central incisor with incomplete obturation and a diffuse radiolucency in relation to the periapical region of central and lateral incisor (Figures 2 and 3).

Endodontic retreatment was initiated in central incisor. Access cavity revealed a restorative material obstructing the access to the main canal. The patient was referred for CBCT (Cone Beam Computed Tomography) scanning to locate the main canal of right central incisor and to study the abnormal root anatomy of maxillary lateral incisor prior to initiating endodontic treatment procedures. The CBCT imaging was done with Kodak 9500 Cone Beam 3D system, Carestream Health Inc., Rochester, NY, USA, with exposure parameters of 90 kVp tube voltage and 10 mA tube current. The images were obtained with a voxel size of 0.20 mm × 0.20 mm × 0.20 mm with exposure time of 10.8 seconds. The images were examined with Carestream 3D Imaging software (Atlanta, GA, USA).

CBCT images of maxillary right central revealed restorative material extending from the cervical to the midroot region labially, suggesting an attempt to repair an improperly directed access opening. The access cavity was reentered; the obstructing cement was removed with GG drill number 3 (Dentsply, Maillefer, Ballaigues, Switzerland) with slow speed drill. The access was refined with CBCT image guidance to locate the main canal. After working length determination with apex locator (Root ZX mini apex locator, J. Morita Corp; Tokyo, Japan) chemomechanical preparation was done with Pro-Taper rotary files (Dentsply Tulsa) up to F3 using 2.5% NaOCl and 17% EDTA as irrigants. Canal projection technique was done using F3 Pro-Taper cone (Dentsply Maillefer) to repair the previous defective access attempt defect with ProRoot MTA (Dentsply Tulsa Dental Specialties, Tulsa, Oklahoma, USA). After the canal was dried with absorbent points (Dentsply, Maillefer) the canal was obturated by lateral condensation technique using 2% gutta percha (Dentsply, Maillefer, Ballaigues, Switzerland) and AH plus root canal sealer (Dentsply De Trey, Konstanz, Germany). The access cavity was restored with composite resin (3M ESPE, St. Paul, MN, USA).

**Figure 4:**
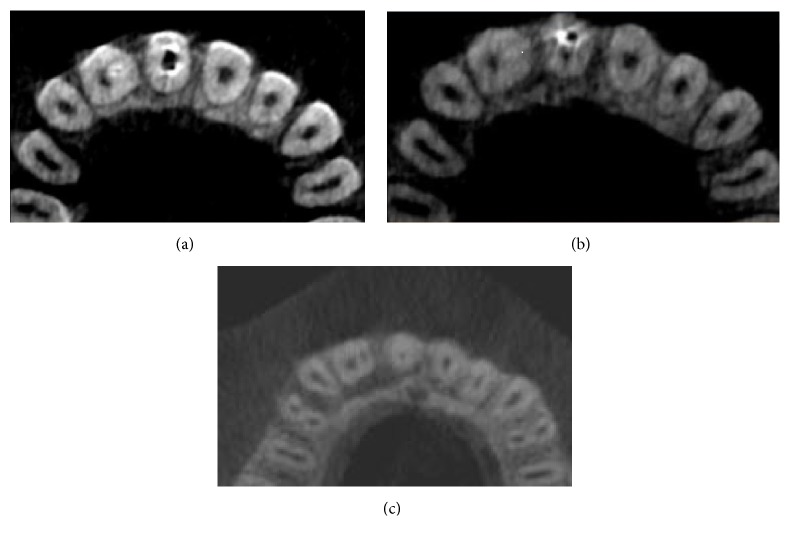
(a) Axial image coronal third of root. (b) Axial image middle third root. (c) Axial image apical third root.

The right lateral incisor showed a continuous main canal and an invaginated portion mesially in the CBCT images. Axial images in CBCT revealed the invaginated tract to be obliterated in the coronal third and midroot area and compressing the main canal (Figures 4(a) and 4(b)).

**Figure 5:**
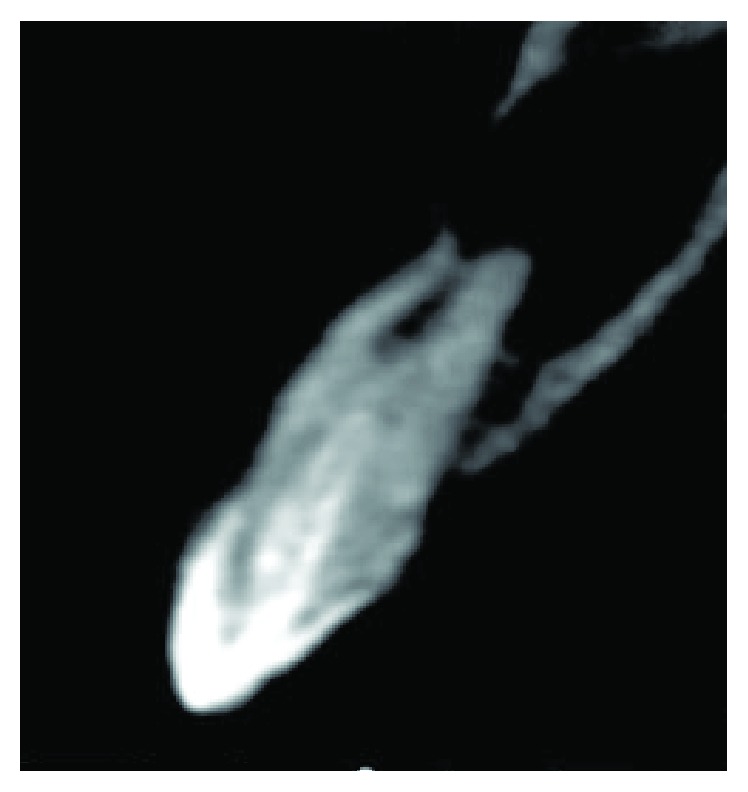
Sagittal view.

The patency of the invagination was observed in the apical third only in the axial and sagittal CBCT images (Figures 4(c) and 5).

Based on these images the case was diagnosed as Oehler's Type III Dens Invaginatus in a nonvital maxillary lateral incisor with periapical lesion. It was planned to do conventional orthograde nonsurgical treatment for the root canal followed by surgical retrograde approach to negotiate and obturate the apical patent portion of the invagination.

CBCT assisted access cavity of lateral incisor was prepared. Access cavity was prepared and the root canal was located distolingually. The orifice of the invaginated portion was not visible. Working length of the canal was determined by apex locater (Root ZX mini apex locator, J. Morita Corp; Tokyo, Japan) and confirmed radiographically with intraoral periapical. The main canal was negotiated and chemomechanical preparation was completed by crown down technique using rotary Pro-Taper Nickel-Titanium Rotary files (Dentsply Maillefer) up to F3 using 2.5% NaOCl and 17% EDTA as root canal irrigants. Calcium Hydroxide was injected in the canal (Calcicur; Voco, Cuxhaven, Germany) as an intracanal medicament for a period of three weeks to promote healing of the periapical lesion. The access was sealed with temporary restoration of Cavit (3M ESPE Norristown; PA, USA).

For the surgical approach, after administration of local anaesthesia using 2% lidocaine with 1 : 1,00,000 epinephrine, a labial full thickness rectangular mucoperiosteal flap was elevated to expose the area of the periapical lesion. The existing pathological cortical bone window was widened with surgical burs to expose the lesion adequately. The granulomatous soft tissue surrounding the root tip was curetted with surgical curettes. Root resection was not done to maintain original root length to help tooth stability.

The main canal of the lateral incisor was obturated by thermoplastic gutta percha backfilling technique using the Elements System (Sybron Endo) of size 30-gauge tip with AH plus as root canal sealer. A retro preparation was done in the apical root portion of the invagination using precurved K files (Dentsply; Mailefer) in a step back manner from number 10 to number 25K file and using 1% NaOCl. The retro preparation was obturated using AH plus (Maillefer, Dentsply, Konstanz, Germany) as sealer and by thermoplastic backfilling with gutta percha (Elements Sybron Endo) using 15-gauge tip and then cold burnished. The coronal access was sealed with glass ionomer cement (Fuji II, GC Corp.). Surgical site was irrigated with normal saline 0.9%, flap repositioned, and sutured with 4-0 nylon suture (Ethicon; Johnson & Johnson). Antibiotics and nonsteroidal inflammatory drugs as well as 2% chlorhexidine mouthwash was prescribed. Patient was called for a follow-up the next day when the soft tissue healing was found to be satisfactory. The sutures were removed after 5 days.

**Figure 6:**
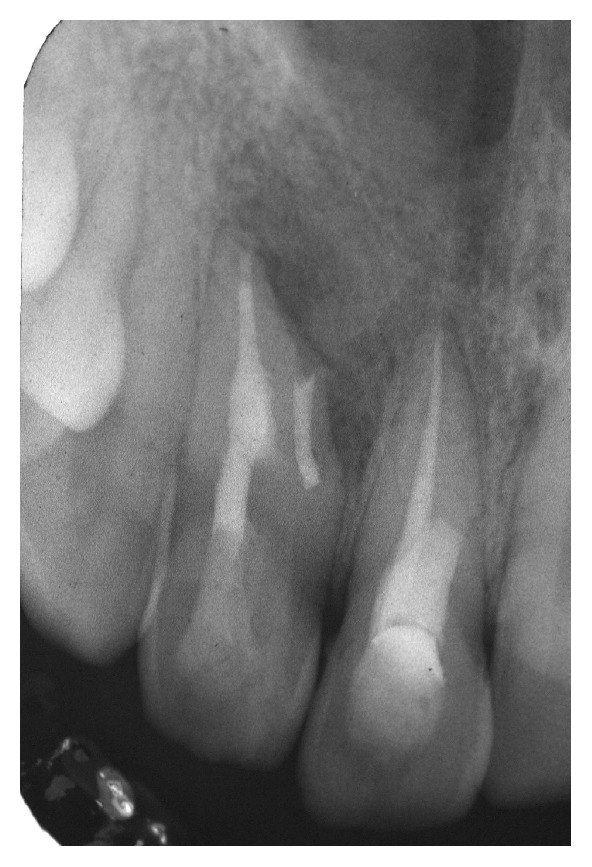
1-year postoperative IOPA.

Follow-up after one year showed satisfactory healing on the radiograph (Figure 6).

## 3. Discussion

Dens Invaginatus (DI) as a dental anomaly shows a broad spectrum of morphologic variations and predisposes the tooth to dental caries and in more severe invaginations to pulpitis and apical periodontitis [2, 3, 8, 12]. Oehler's Type III Dens Invaginatus as reported in this case is considered as the most severe variant of the anomaly wherein the affected tooth shows a deep infolding of the enamel and dentin extending into the root and communicating laterally with the periodontal space through a pseudo foramen [1, 8].

The enamel lining in these defects is often hypomineralized and incomplete with various channels that communicate with the pulp thereby allowing easy access for irritants from the invagination to reach the pulp space [12, 14].

Varied treatment options for Oehler's Type III DI have been mentioned in the literature. Suggested treatment modalities range from periodic observation to prophylactic sealing of the invaginated pit in asymptomatic teeth. In cases with pulp necrosis, endodontic therapy of only the invaginated portion [4] or of both the invaginated portion as well as the root canal has been suggested [15]. Surgical endodontics in addition to conventional endodontics should be considered only for large periapical lesions not resolved nonsurgically or in cases which cannot be treated nonsurgically due to failure to gain a coronal access. Surgery will provide a disinfection and retrograde seal to the apical portion of the invagination and/root canal which will otherwise retain a source of infection [2, 5–7, 9, 10, 16–18]. The surgical endodontic procedures include apical resection, apical curettage, and retrograde restoration. Extraction of the teeth is proposed only for those teeth that cannot be salvaged surgically [2, 4, 6, 7, 9, 14, 15]. Complete removal of the invaginated portion to create a large canal space as well as intentional replantation to treat Type III DI has been reported in literature [2, 9].

Type III DI is often associated with changes in the morphology of the root canal itself; thereby endodontic procedures are a challenging task due to the aberrant anatomy in both the pulp space and the invaginated track. Also, there can be an absence of a true apical constriction if the invagination opens into the periodontal space (as reported in this case) (Figure 5) which further contributes to making endodontic procedures complicated [2, 3, 7, 9, 15, 17]. The invaginated portion may compress the main canal at different levels as reported by Fregnani et al. [16] and as was seen in CBCT images in this case report (Figures 4(a) and 4(b)).

Cases of DI with its complex anatomy always pose a diagnostic and treatment challenge to the clinician. Due to the intrinsic limitations of the conventional radiographs, they are unable to reveal the details of the DI three-dimensionally. The introduction of CBCT in the 1990s with its 3D images has revolutionized the imaging of dental malformations and contributes in a large way to diagnosis and treatment planning of these cases. In cases of DI, CBCT images show the details in terms of type, extension, and morphologic changes in the tooth [7, 13, 19, 20]. CBCT uses low effective radiation dose and besides generating undistorted 3D reconstruction of the teeth and surrounding soft tissues provides interrelational images in three orthogonal planes: axial, sagittal, and coronal [21, 22].

We have presented a challenging case of Oehler's Type III DI in a maxillary lateral incisor associated with necrotic pulp and periradicular lesion. The use of Cone Beam Computed Tomography (CBCT) images was invaluable in diagnosis and treatment planning. The CBCT axial images revealed that in the coronal third of root the invagination was nearly obliterated and compressing the main canal outline (Figure 4(a)). In the midroot region the invaginated portion was partially patent towards the apical end while the apical portion of the invagination was wide open at its exit portal (Figure 5). As compared with the contralateral incisor the larger cross section of the invaginated tooth was evident (Figures 4(a), 4(b), and 4(c)). The invaginated canal seemed to be lined with enamel in the coronal and midroot region (Figure 5). Based on these CBCT images we decided to treat the main root canal with nonsurgical endodontic therapy and do a surgical endodontic approach with retrograde filling for the patent apical portion of the invagination (Figures 4(c) and 5). Due to the risk of lateral perforation with burs, conventional access to the obliterated portion of the invaginated portion was not made, nor was an attempt to join the invagination with the root canal done. A similar combination of conventional and surgical endodontics has also been reported in a case report by Vier-Pelisser et al. [7].

Calcium Hydroxide was used as an intracanal medicament in the root canal for a period of two weeks as its high alkalinity facilitates healing in necrotic teeth with periapical lesion.

Even though MTA has been the preferred root end material, our choice of gutta percha for the orthograde and retrograde obturation was based on its ability to be heat softened thereby allowing it to penetrate all parts of the aberrant canal anatomy of the DI (Figure 6). The advantage of the thermoplasticized gutta percha obturation has been well documented for both orthograde and retrograde fillings [2, 10, 15, 18].

The use of the CBCT images in this case was important in treatment planning and for successfully treating a case of Oehler's Type III DI with nonvital pulp and periapical lesion with combined nonsurgical and surgical endodontic therapy.

## 4. Conclusion

Teeth with Oehler's Dens Invaginatus Type III and with periapical pathosis are a challenge to the endodontist considering their aberrant anatomy. The use of CBCT was an important tool in our case to provide relevant details of the internal anatomy of the invaginated tooth and thereby played a vital role in treatment planning. The combination of nonsurgical and surgical treatments was an effective approach in our case.
